# Female genital mutilation — a blind spot in Dutch general practice? A case–control study

**DOI:** 10.3399/bjgpopen20X101105

**Published:** 2020-12-02

**Authors:** Ramin Kawous, Nigar Kerimova, Maria ETC van den Muijsenbergh

**Affiliations:** 1 Department of Public Health, Erasmus University Medical Centre, Rotterdam, The Netherlands; 2 Pharos, Dutch Centre of Expertise on Health Disparities, Utrecht, The Netherlands; 3 Department of Primary and Community Care, Radboud University Medical Centre, Nijmegen, The Netherlands

**Keywords:** female genital mutilation/cutting, general practice, migrant women, primary health care, culturally competent care

## Abstract

**Background:**

Women with female genital mutilation or cutting (FGM/C) often suffer from physical and psychosexual problems related to FGM/C. As gatekeepers to the medical system, GPs are often the first to be consulted about these problems. It is as yet unknown if, and to what extent, Dutch GPs identify women with FGM/C or related health problems.

**Aim:**

To investigate how often Dutch GPs register FGM/C and related health problems.

**Design & setting:**

A case–control study of anonymised patient records was performed in the Netherlands.

**Method:**

Medical records were checked for information on country of origin. Records of women, aged ≥15 years, from countries where FGM/C is practised were compared with those of a case-control.

**Results:**

Although many migrants were registered with the participating GPs, information on country of origin was seldom recorded. Only 68 out of 16 700 patients were identified as women from countries where FGM/C is practised; 12 out of these 68 records contained information about the FGM/C status, but none on the type of FGM/C. There were no significant differences in health problems related to FGM/C between patients with FGM/C and the controls.

**Conclusion:**

FGM/C may be a blind spot for GPs and registration of information on migration background could be improved. A larger sample in a future study is needed to confirm this finding. Given the growing global migration, awareness and knowledge on FGM/C, and other migration-related health issues should be part of GP training.

## How this fits in

The majority of previous research has focused on FGM/C in maternity settings. There is a need for more research on FGM/C in primary care such as general practice.^1^ This is the first study to investigate the provision of health care to women with FGM/C in Dutch general practice.

## Introduction

FGM/C consists of *‘all procedures that involve partial or total removal of the external female genitalia or other injury to the female genital organs for non-medical reasons* *’*.^2^ The World Health Organization (WHO) has classified four main types of FGM/C: type I, or clitoridectomy, refers to excision of the prepuce, with or without excision of part or all of the clitoris; type II, or excision, which consists of the clitoris with partial or total removal of the labia minora; type III, the most severe form of FGM/C, which is known as infibulation, refers to excision of part or all of the external genitalia and stitching or narrowing of the vaginal opening. Type IV is unclassified. It includes all other harmful procedures to the female genitalia for non-medical purposes; for example, pricking, piercing, incising, scraping, and cauterising the genital area.^3^

FGM/C is predominantly practised in countries in Africa, the Middle East, and Asia. Globally, it is estimated that more than 200 million girls and women have undergone FGM/C,^4^ and that an estimated 68 million girls are at risk of being cut between 2015 and 2030.^5^ As a result of increasing migration of women from countries where FGM/C is concentrated, FGM/C has become common in high-resource countries such as the Netherlands. It is estimated that approximately 41 000 girls and women with FGM/C are living in the Netherlands. About 37% of these girls and women are estimated to have been infibulated.^6^

FGM/C is recognised as a human rights violation^2^ and many countries have increasingly undertaken law reform to prohibit FGM/C.^7^ In the Netherlands, performing any form of FGM/C is forbidden and punishable by law, with an incarceration term of up to 12 years. The law also prohibits any Dutch nationals or permanent Dutch residents from performing FGM/C abroad.^8–10^ However, there is no indication of FGM/C being performed in the Netherlands and no suspected FGM/C case has, as yet, been convicted by a court.^11^

The Dutch action plan against the practice of FGM/C was established in 2006. The rationale behind the Dutch approach against FGM/C is to prevent FGM/C by accomplishing a behavioural change towards the practice. The prevention policies in the Netherlands consist of, among others, awareness raising on FGM/C among communities concerned and the empowerment of women, as well as training of health professionals and healthcare workers to support women affected by FGM/C, and to identify and prevent girls from being cut.^12^ Healthcare providers, social care professionals, and teachers who are concerned that a girl may be at risk of FGM/C have legal obligation to report this.

Recently, the Dutch Society of Obstetrics and Gynaecology (NVOG) has developed guidelines on the management of FGM/C.^13^ Healthcare providers in primary and secondary health care are also able to record FGM/C and they have been urged to practise good record-keeping. Unfortunately, to date, no reliable data are available in the Netherlands regarding FGM/C in primary and secondary care.

FGM/C has been associated with adverse short- and long-term health consequences, including, severe pain, excessive bleeding, urinary tract infections, bacterial vaginosis, painful sexual intercourse, and adverse perinatal outcomes,^14–16^ as well as negative effects on mental health.^17–19^ The migration from countries where FGM/C is practised to high-resource countries will further increase.^20,21^ Consequently, in the near future, healthcare providers are expected to provide care for more women with FGM/C. There is increasing evidence that women with FGM/C receive lower quality of care, owing to *‘lack of knowledge, cultural sensitivities associated with the subject leading to silence, stigma and inaction* *'*.^1^ Since most of these studies have been conducted in obstetric settings, ^22–25^ there is a need for more research on FGM/C in primary care, such as in general practice.

In the Netherlands, the GP is often the first to encounter health problems of women with FGM/C. GPs function as a ‘gatekeeper’ to the healthcare system in the Netherlands and all residents are obliged by their (mandatory) health insurance to be registered with a general practice. However, it is as yet unknown if, and to what extent, GPs identify women with FGM/C, nor what care they provide to them. Insight into these aspects are vital in order to evaluate whether any inequities exist in primary health care for women with FGM/C. The aim of this study was to investigate how often in Dutch general practice FGM/C-related health problems were recorded in women from countries where FGM/C is practised, compared with women with other migration background of the same age.

## Method

### Study design

A case–control study of anonymised patient records of women with a migration background, including women from countries where FGM/C is practised, was carried out between November and December 2017.

### Recruitment and study population

The study was executed in five general practices in the Netherlands, known to care for a large migrant community and trained to care for women with FGM/C. In the Netherlands, it is becoming more difficult to encourage GPs to participate in surveys, owing to their demanding work schedules and increasing frequency of being approached for surveys.^26^ Therefore, the practices were recruited through the researcher's informal network. A purposive sampling method was used, aiming for practices with a high number of patients from very high FGM/C prevalence countries, striving for variation among GPs in terms of sex, size, and geographical location of the general practice.

Patient records of girls and women, aged ≥15 years, from very high FGM/C prevalence countries, including Somalia, Egypt, Eritrea, Sierra Leone, and Sudan, were included. The Netherlands has a large number of migrants from these countries in which more than 80% of girls and women of reproductive age have undergone FGM/C. Since country of origin is not routinely registered in general practice, all female patients with a foreign surname were first included. Identifying migrants by means of their surname has proved to be a reliable, second-best method.^27^ Next, information on country of origin was searched through the first 10 contact records, including the first visit records. Then, notes were searched to retrieve information on country of origin, using the keywords 'origin', 'country of birth' or 'country of origin', 'Somalia', 'Eritrea', 'Egypt', 'Sudan', or 'Sierra Leone'. The data of these women were compared with those of a case-control: female migrant patients in the same age group from countries where FGM/C is not practised. Every next migrant woman from a non-FMG/C practising country was selected from the same age-group on the list after each woman from a country where FGM/C is practised was selected.

It has been well documented that migrants, in general, have at some point differing morbidity patterns and receive lower quality of care than native populations.^28^ Hence, within the scope of this study, it was found to be more appropriate to compare the data within migrant subgroups, so data of women from countries where FGM/C is practised were compared with female migrant patients from countries where FGM/C is not practised.

### Data collection

The researcher (NK), a senior medical student, was allowed to access the complete patient records in each participating general practice, after signing the required confidentiality agreement, in line with national legislation on data protection. NK selected the records based on patients’ surnames and then anonymised the data by converting each patient’s personal details into an alphanumeric code. Records contained information on every contact with the GP (in person or by telephone), medication prescribed by the GP, and all letters to the GP from other (health) professionals and services.

It was checked whether country of origin, FGM/C status of the patient, type of FGM/C, or health problems often associated with FGM/C were recorded in medical files. The files were searched for health problems, including recurring urinary tract infections (defined as two or more cystitis in 6 months), dysmenorrhoea, sub- or in-fertility, complications during childbirth, painful sexual intercourse, and fear of sexual intercourse. In addition, it was checked whether the FGM/C status of the patient was recorded according to the International Classification of Primary Care (ICPC). The ICPC code-X82 refers to injury to the female genital organs. Finally, women with unknown origin were excluded and the data were anonymised prior to analysis.

### Data analysis

The data were analysed using SPSS statistical software (version 22). The χ^2^ test was used to compare the data from different groups and to determine whether the differences were statistically significant.

## Results

A total of 16 700 patient records from five general practices were included in the analysis. Country of origin of patients from FGM/C-prevalent countries was mentioned in 68 cases. As shown in Table 1, these women were from (in descending order) Somalia (58.8%), Sudan (13.2%), Egypt (11.8%), Eritrea (10.3%), and Sierra Leone (5.9%), with an average age of 36.18 years (standard deviation [SD] = 14.76). The majority of women in the control group were from Morocco (63.9%), with an average age of 39.82 years (SD = 13.52).

**Table 1.** Number of women in the FGM/C and control groups in five general practices in the Netherlands

**Control group** **(*n* = 61)**	*n*	**FGM/C group** **(*n* = 68)**	*n*
**Country of origin**			
Afghanistan	2	Egypt	8
Armenia	3	Eritrea	7
Azerbaijan	1	Sierra Leone	4
China	3	Somalia	40
Morocco	39	Sudan	9
Pakistan	1		
Syria	1		
Taiwan	2		
Turkey	7		
Vietnam	2		
**Mean** **age** **,** **years**	39.82		36.13
SD	13.52		14.76
Range	18–74		15–74

FGM/C = female genital mutilation or cutting; SD = standard deviation

The FGM/C status of patients was recorded in 12 out of the 68 cases where country of origin was documented. Of these patients, 11 had undergone some form of FGM/C. In none of these cases was type of FGM/C classified and recorded. Moreover, the FGM/C status of one patient was recorded using the ICPC code-X82. Other cases were randomly recorded or found in the correspondence.

There were no significant differences in recorded health problems related to FGM/C such as urinary tract infections, dysmenorrhoea, sub- or in-fertility, complications during childbirth, painful sexual intercourse, or fear of sexual intercourse between patients with FGM/C (*n* = 12), patients whose FGM/C status was not recorded (*n* = 56) and patients in the control-group (*n* = 61).

## Discussion

### Summary

In this first study on FGM/C in five general practices in The Netherlands known to care for a large migrant community, patient demographics such as country of origin were seldom documented in the medical charts. Only 68 out of 16 700 patients were identified as women from very high FGM/C prevalence countries; 12 out of these 68 medical files contained information about the FGM/C status of the patient, and in none of these cases was type of FGM/C classified.

Although the participating practices were known to care for a large migrant community and trained to care for women with FGM/C, it is unclear why information on country of origin was seldom recorded and only 12 records contained information about FGM/C. It is believed that it is highly unlikely that only 12 out of 68 women from very high FGM/C prevalence countries had actually undergone FGM/C. FGM/C may be a blind spot in general practice in the Netherlands. Future research, preferably qualitative, is needed with larger samples to confirm this, and to explore the factors that encourage and hinder GPs in recording and discussing FGM/C with women who have undergone the procedure.

### Strengths and limitations

The relatively small numbers of general practices included in the study is acknowledged, which may explain the non-significant difference in recorded health problems related to FGM/C between patients in the FGM/C group and patients in the control group. While healthcare providers are able to systematically record FGM/C, to date, no reliable data are available regarding FGM/C in primary and secondary care. Therefore, the authors believe that it may not be necessary to include more practices before concluding that there is an urgent need for improvement of recognition and recording of FGM in general practice; improvement could be achieved by discussing FGM/C with patients from FGM/C-prevalent countries, and by correctly classifying and recording FGM/C in the medical charts.

### Comparison with existing literature

There is limited evidence on FGM/C in primary care. Most studies have been conducted in secondary care and have focused on FGM/C in maternity settings.^1^ Nonetheless, the present findings would be consistent with results from available studies among other healthcare providers caring for women with FGM/C in secondary care.^29^ Several reasons may explain the few recorded cases of FGM/C in general practice. In general, it is known that most women rarely proactively discuss FGM/C with their healthcare provider. At the same time, healthcare providers refrain from asking about it.^1,17,30–33^ The possible explanation could be owing to cultural taboos around FGM/C in women’s own community or fear of being judged, or being fearful as FGM/C is forbidden in their new country of residence.^34^

In a comprehensive systematic review, Evans *et al* ^1^ have explored factors influencing the provision of health care related to FGM/C from the perspective of health providers. Feeling of embarrassment, shock, disgust, and horror; uncertainty about how to frame the questions, or *‘* *anxiety about being perceived as culturally insensitive* *’*, language barriers, and a lack of knowledge have been described as barriers to engagement with women with FGM/C.^1,22–25,30,32,33,35–43^

Because of the sensitive nature of FGM/C, talking about the subject can make health providers feel uncomfortable. Some healthcare providers reported they avoided discussing the topic with their patient, as they did not want to offend, stigmatise, or jeopardise their relationships, assuming that women would initiate a conversation about FGM/C if there was a problem.^1^ As a result, FGM/C will probably never be discussed at all, hence not recorded. As mentioned, previous studies have reported expression of strong emotions by healthcare providers, including shock, disgust, and horror. Cutting of women was perceived as an alien and negative practice, with healthcare providers describing and perceiving these women as not ‘normal’, but mutilated. Also, lack of knowledge has been listed repeatedly as a barrier to identify and manage women with FGM/C.^1,22–24^ For instance, Chalmers *et al* ^22^ reported dissatisfaction of women with care and a perceived lack of knowledge and ability by healthcare providers to care for women during pregnancy and childbirth. Relph *et al* ^23^assessed the knowledge, attitude, and training on FGM/C among medical and midwifery professionals and found that although the majority of these professionals were aware of the practice, their ability to identify FGM/C and its associated health complications remains unsatisfactory. Similarly, Zaidi *et al* ^24^ observed deficiencies in knowledge and adherence to guidelines on the management of health complications of FGM/C among healthcare providers. Zenner *et al* ^25^ reported on the quality of obstetric and midwifery care, and noted major deficits in identification, management, and safeguarding.

### Implications for research and practice

The findings, along with those of previous reports, support the need for integration of FGM/C and culturally sensitive care in training programmes for healthcare providers. Currently, despite the growing number of books on migration health in primary care,^44–47^ the subject of FGM/C is not included in the curriculum of most medical, midwifery, and public health training programmes.^48,49^ In addition, specific guidelines on the management of health complications related to FGM/C are not well known among healthcare providers.^48^

As noted by Abdulcadir *et al*,^29^ recognition of FGM/C is the first step in formulating a diagnosis, before providing information and treating a patient with FGM/C. However, FGM/C may be a blind spot in general practice in the Netherlands. Future research, preferably qualitative, is needed with larger samples to confirm this and to explore the factors that encourage and hinder GPs in recording and discussing FGM/C with women who have undergone it.

Further, registration of information on migration background could be improved and, as migration is on the rise and the population attending general practices gets more and more diverse, GPs should not only be aware and knowledgeable on FGM/C, but also on other migration-related health issues.
